# Machine learning evaluation of a hypertension screening program in a university workforce over five years

**DOI:** 10.1038/s41598-024-74360-1

**Published:** 2024-12-04

**Authors:** Olumide Adeleke, Segun Adebayo, Halleluyah Aworinde, Oludamola Adeleke, Abidemi Emmanuel Adeniyi, Oluwasegun Julius Aroba

**Affiliations:** 1 Directorate of Health Services, Bowen University, Iwo, Nigeria; 2 College of Health Sciences, Bowen University, Iwo, Nigeria; 3 College of Agriculture, Engineering and Science, Bowen University, Iwo, Nigeria; 4 College of Computing and Communication Studies, Bowen University, Iwo, Nigeria; 5 Honorary Research Associate, Department of Operations and Quality Management, Durban University of Technology, Durban, South Africa; 6 Centre for Ecological Intelligence, Faculty of Engineering and the Build Environment (FEBE), Electrical and Electronic Engineering Science, University of Johannesburg, Auckland Park Campus, Auckland Park, P.O. Box 524, Johannesburg, 2006 South Africa

**Keywords:** Machine learning, Artificial Intelligence, Hypertension/diagnosis, Hypertension/prevention & control, Screening programmes, Occupational health, Medical records systems, workplace, Biomedical engineering, Mathematics and computing, Computer science

## Abstract

The global prevalence of hypertension continues excessively elevated, especially among low- and middle-income nations. Workplaces provide tremendous opportunities as a unique, easily accessible and practical avenue for early diagnosis and treatment of hypertension among the workforce class. The evaluation of such a Workplace Screening Strategy can give insight into its possible effects. Innovative machine learning approaches like k-means clustering are underutilized for such assessments. We set out to use this technology to analyze the results of our university’s yearly health checkup of the employees for hypertension. An anonymized dataset including the demographics and blood pressure monitoring information gathered from workers in various departments/units of a learning organization. The overall amount of samples or data values is 1, 723, and the supplied dataset includes six attributes, such as year group (2018, 2019, 2021, 2022), Department/Unit (academic and non-academic), and gender (male and female), with the intended output being the blood pressure status (low, normal, and high). The dataset was analyzed using machine learning approaches. In this longitudinal study, it was discovered that the average age for the workforce is 42. Similarly, it was revealed that hypertension was common among employees over the age of 40, regardless of gender or occupational type (academic or nonacademic). The data also found that there was a consistent drop in the prevalence of hypertension from 2018 to 2022. According to the study findings, the use of machine learning algorithms for periodic evaluations of workplace health status monitoring initiatives (particularly for hypertension) is feasible, realistic, and sustainable in diagnosing and controlling hypertension among those in the workforce.

**Supplementary Information:**

The online version contains supplementary material available at 10.1038/s41598-024-74360-1.

## Introduction

Hypertension, simply known as elevated blood pressure, is the number one risk factor for death globally, significantly increasing the risk of developing cardiovascular, brain, and kidney diseases^1^. Globally, 1.28 billion people between the ages of 30 and 79 were reported to have hypertension in 2019. Two-thirds of these adults reside in low- and middle-income nations^2^. Regrettably, the WHO African Region has the highest prevalence of hypertension (27%) while the WHO Americas Region has the lowest prevalence of hypertension (18%)^2^. Sadly, an estimated 46% of persons with hypertension are ignorant of their disease and only 21% of persons with hypertension have it under control^3^. Screening programmes for hypertension could help to reduce morbidity and mortality associated with the condition in adults^4^.

Employee’s health status is of great importance to the stability and development of any institution and the society at large. However, it has been reported that the cardiovascular health status of the occupational population worldwide is not optimal^5^. Fortunately, workplace health programmes such as employee’s periodic screening have been shown to protect the individual employee’s overall health^6^, improve his/her productivity^7^, lower the overall medical costs^7^, and reduce disease prevalence in general^8,9^. Occupational screening of employees at work is an effective approach of identifying undiagnosed hypertensive people^10^.

Artificial intelligence (AI) has been used in many areas of healthcare services, including medical imaging and diagnostics, pandemic management, virtual patient care, promoting medication and vaccine innovation, lowering the administrative burden on medical staff, tracking patient adherence to treatment regimens, conducting gait analyses for technology-assisted rehabilitation, and increasing patient engagement^11^. AI has also been used in some published research to diagnose^12,13^ and to forecast the prevalence of hypertension^14,15^ in the general population. However, machine learning approaches such as k-means clustering are underutilized in the context of workplace screening. Leveraging machine learning in workplace offers a promising opportunity for advanced data analysis techniques to enhance health assessments in the workplace.

K-Means Clustering is a machine-learning technique capable of providing useful insight into the behavior and patterns of data^16^. It has been applied in diverse areas in medical science such as cancerous cell detection, segmentation of brain images, skin treatment, intrathoracic airway trees, and abnormality detection of heart ventricles^17,18^. Analysis of workplace and occupational screenings, where patient data may be complex, multidimensional, and subject to variable degrees of uncertainty, is particularly well-suited for k-Means clustering. In this study, we applied the k-Means Clustering algorithm to analyze health medical records from the university workforce^19^.

## Methodology

### Study site and participants

This retrospective study was carried out between December 17, 2018, and December 20, 2022, at the Bowen University Hospital located in the urban setting of Iwo (population range of 250,000-499,999 inhabitants), Osun State in southwestern Nigeria. Bowen University, founded by the Nigerian Baptist Convention in 2001, is one of the oldest private coeducational institutions of higher education in Nigeria. This retrospective study included 1723 rows of datasets from the workforce at Bowen University across different academic and non-academic units of the university.

### Dataset acquisition

The dataset was collated from staff in different units of an academic institution for a period of four years (2018, 2019, 2021 and 2022). The blood pressure measurements used in this screening programme were taken by nurses and community health extension workers trained in manual blood pressure measurement, including proper body positioning during measurement. BPs were measured manually using a stethoscope and the appropriate sized brachial pressure cuff with a sphygmomanometer. The participants did not smoke or ingest caffeine or other stimulants or food in the 30 min before the measurements, which were taken after at least 5 min of rest in a quiet and calm environment Two BP readings were taken on both arms of employees in a sitting position and at 2-minute intervals. If the first two readings differ by more than 10mmHg, additional readings were obtained in line with American Heart Association (AHA) recommendations^20–22^.

Equipment was inspected on a routine basis to ensure accuracy. Employees with measurements outside of the recommended range during the screening programme were advised to follow up with medical doctors at the Bowen University Hospital. High blood pressure (hypertension) at the time of screening is defined as a systolic blood pressure of 140 mmHg or higher or a diastolic blood pressure of 90 mmHg or higher National Heart, Lung, and Blood Institute^23^. We then anonymized these data from the university’s yearly health checkup to ensure privacy while providing valuable insights into the prevalence of hypertension among different demographic groups.

### Data preprocessing

The dataset needs to be pre-processed before model training because each input sample contains different features with missing and inconsistent values. Most of the missing data in the dataset are measured values from staff, therefore, the study employed data-cleaning techniques. This entails locating and fixing mistakes or discrepancies in the data, including duplicates, outliers, and missing numbers^24,25^. The data cleaning was accomplished with a variety of methods, including imputation, removal, and transformation.

### Data correlation

Data mining task play an important role in discovering patterns in data. This study employed feature correlation and k-means clustering to find relevant and non-redundant features in the data. Pearson correlation coefficient was employed to understand the linear relationships between variables. The Pearson Correlation Coefficient between two variables ***X*** and ***Y*** is computed as:

1 $$\:{\rho\:}_{\left(X,Y\right)}=\frac{COV(X,Y)}{{\sigma\:}_{X}{\sigma\:}_{Y}}$$

Where *cov is the covariance*, $\:{{\upsigma\:}}_{\text{X}}\:$*is the standard deviation of variable X and*$\sigma _{Y} {\text{is}}\:{\text{the}}\:{\text{standard}}\:{\text{deviation}}\:{\text{of}}\:{\text{variable}}$*Y*.

### Data clustering

K-means clustering algorithms^30^ are used to discover the structure of data and form the cluster. This is achieved by dividing the dataset into clusters according to data similarity. The technique involves initially selecting ‘k’ features randomly from original dataset D, as initial cluster centres. Based upon the distance between the features and cluster mean, the most similar object is assigned to the cluster. New mean value is then calculated for each cluster. The latter step is repeated until there is no redistribution of features in any cluster.

### Ethical consideration

This study was conducted using de-identified data obtained from the health medical record unit of the University Hospital. Ethical approval was sought and obtained from the Directorate of Research and Strategic Partnerships of the University.

## Results

### Initial dataset exploration

The dataset contains both sociodemographic and medical records. The data set is summarized in Table 1 based on different features. The dataset contains seven features which are stated as year, age, systolic and diastolic values, blood pressure status, department or unit, and gender. There are 1723 rows in the dataset and the mean age is 42.64 years old, mean systolic and diastolic measurement are 120.91 and 78.4 respectively.

The dataset contains bio and blood pressure measurements values obtained from staff of different departments/Unit in an academic institution. The total number of samples or data points are 1, 723 in which the input dataset contains six features, including year category (2018, 2019, 2021,2022), Department/Unit (academic and non-academic), gender (male and female), while the target output is the blood pressure status (low, normal and high) respectively.

**Table 1:** Summarized dataset.

Statistic	Year	Age	Systolic_Blood_Press	Diatolic_Blood_Press	Blood_Press_Status	Dept_Unit	Gender	Category
count	1723	1723	1723	1723	1723	1723	1723	1723
unique	NaN	NaN	NaN	NaN	3	239	2	2
top	NaN	NaN	NaN	NaN	Normal	Registry	M	Non-academic
freq	NaN	NaN	NaN	NaN	1237	288	891	1172
mean	2019	42.6	120.9	78.4	NaN	NaN	NaN	NaN
std	1.3	9.7	18.6	23.1	NaN	NaN	NaN	NaN
min	2018	17	80	10	NaN	NaN	NaN	NaN
25%	2018	35	110	70	NaN	NaN	NaN	NaN
50%	2019	42	120	80	NaN	NaN	NaN	NaN
75%	2019	50	130	80	NaN	NaN	NaN	NaN
max	2022	76	230	910	NaN	NaN	NaN	NaN

**Fig. 1:**
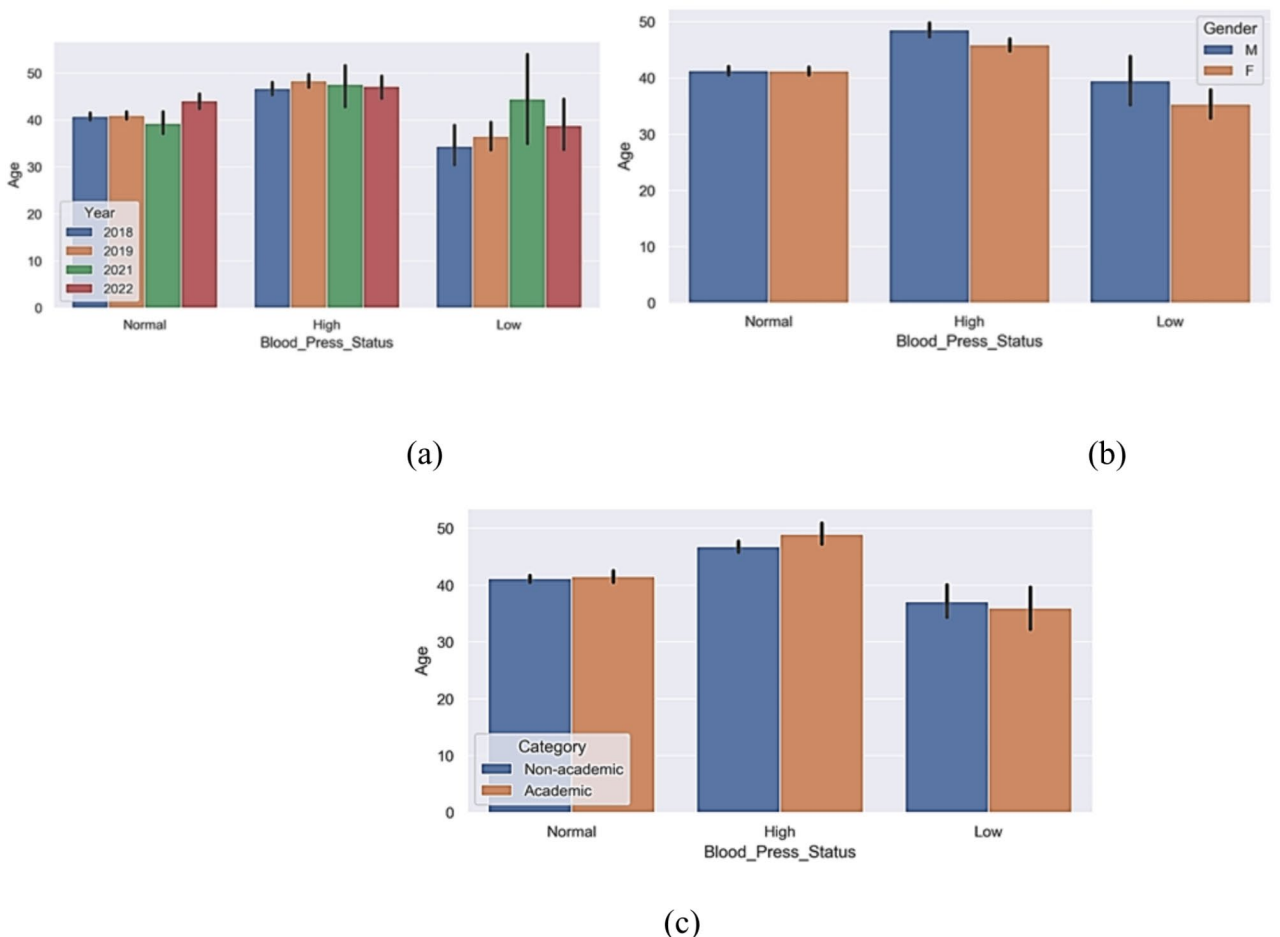
Blood status against age across different (**a**) years, (**b**) gender, (**c**) category.

The number of individuals with normal blood pressure is higher than low or high blood pressure as shown in Fig. 1. The mean for systolic blood pressure value is about 124. Insight from Fig. 1a and b show that high blood pressure is prevalent among staff above the age of 40 irrespective of their gender or category (academic or non-academic).

**Fig. 2:**
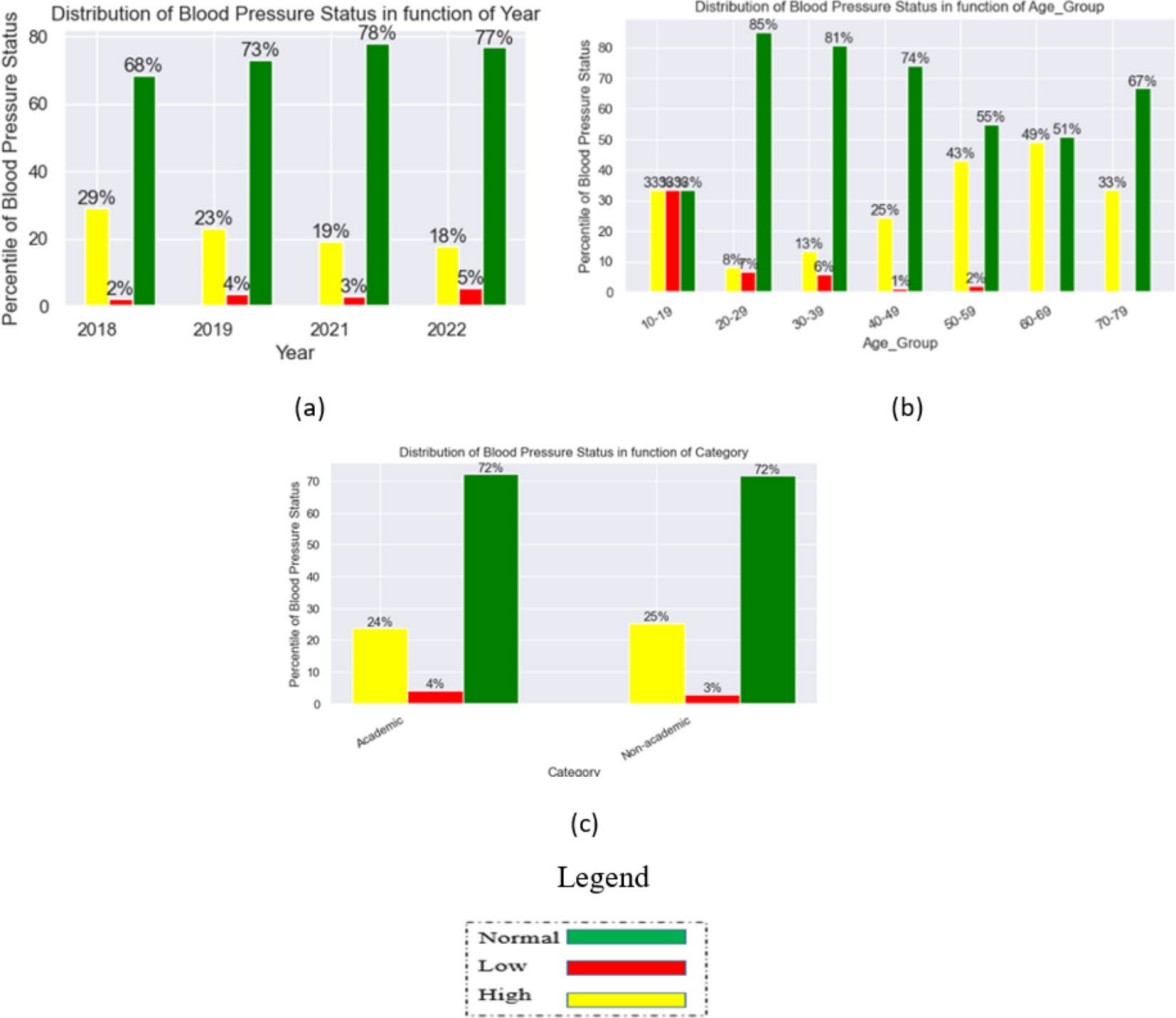
Relationship plots of the variables in the dataset: (**a**) Distribution of blood pressure status in function of year; (**b**) Distribution of blood pressure status in function of Age-group; (**c**) Distribution of blood pressure status in function of categories.

Figure 2 shows the relationship plots of the dataset variables. The graphs revealed that there has been a relative decline in high blood pressure between 2018 and 2022. As against 29% high blood pressure in 2018, there has been a relatively significant fall to 18% in 2022 which accounts for 11% improvement in healthcare of individual staff by reason of blood pressure. Conversely, there seems to be a slight rise (3%) in low blood pressure from 2% in 2018 to 5% in 2022; this may be another trend to really pay attention to and the attendant contributive factors.

From the age-range variable, high blood pressure is prevalent among the age range 60–69 with 49% prevalent rate while the age group 20–29 has the lowest rate of 8%. In terms of low blood pressure, the age group 20–29 has the highest rate of 7% while the age group 40–49 has the lowest rate in low blood pressure of 1%.

For the staff category, the difference in high blood pressure rate is not so significant as there is only a difference of 1% between academic and non academic staff. Non academic staff members have a high blood pressure rate of 25% while the academic category of staff has 24%.

### Features correlation

**Fig. 3:**
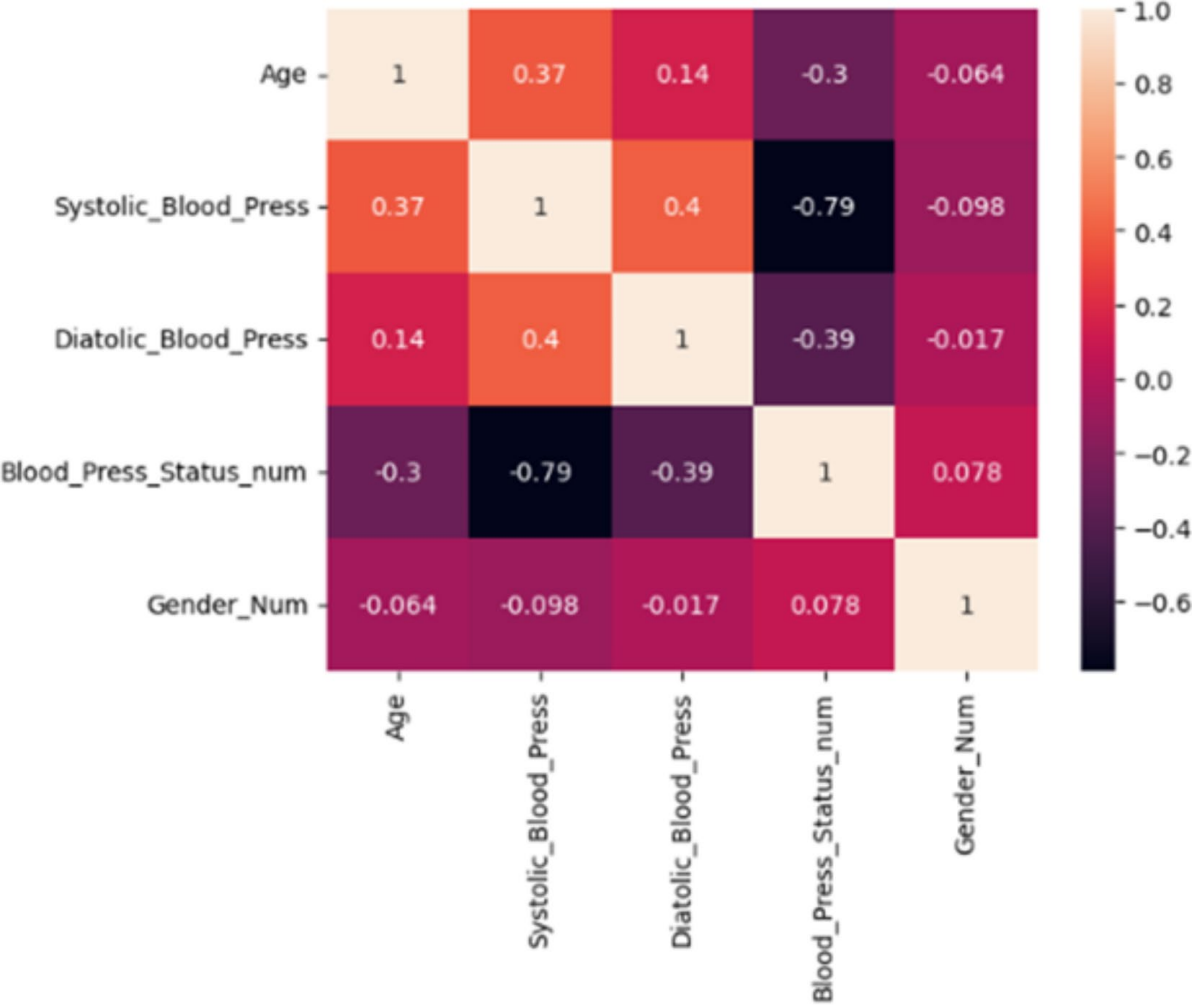
Pearson Correlation Matrix of the Variables in the dataset.

**Fig. 4:**
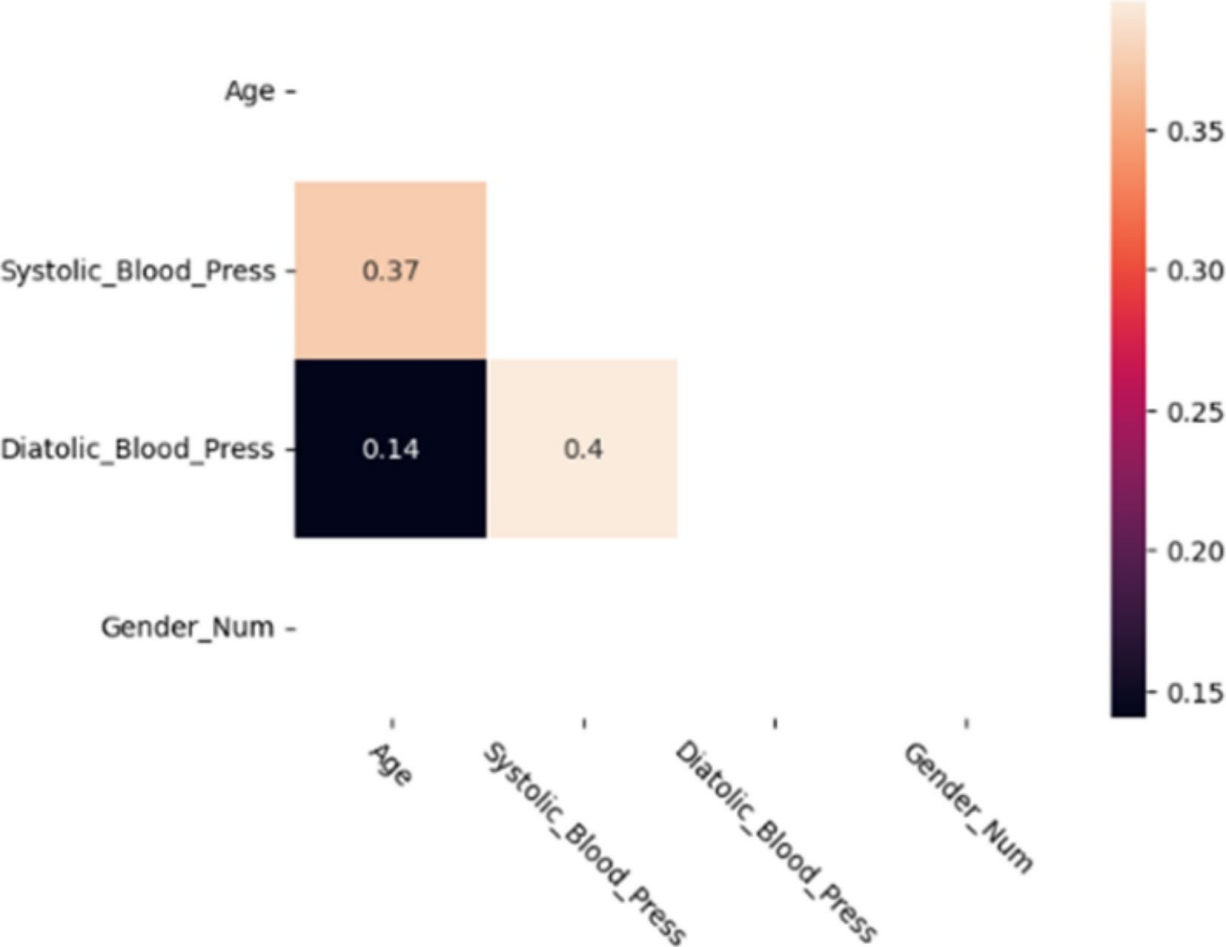
Pearson Correlation Matrix of the Variables in the dataset with non-significant values and the upper triangular filtered out.

After looking at the trends for the main variables in the previous section, the study considered the potential correlations between various variables. Figure 3 shows the Pearson Correlation matrix with significant values obtained from the dataset. Significant values have been identified as values with a 95% confidence and different from zero^29^. However, studies have shown that variable correlation can be full of noise or misrepresentation. To prevent such in our results, the study performed t-test^30^ and the result is presented in Fig. 4. This shows that there is a strong correlation between systolic and age as well as diastolic and age.

### Insight through unsupervised learning: clustering

**Fig. 5:**
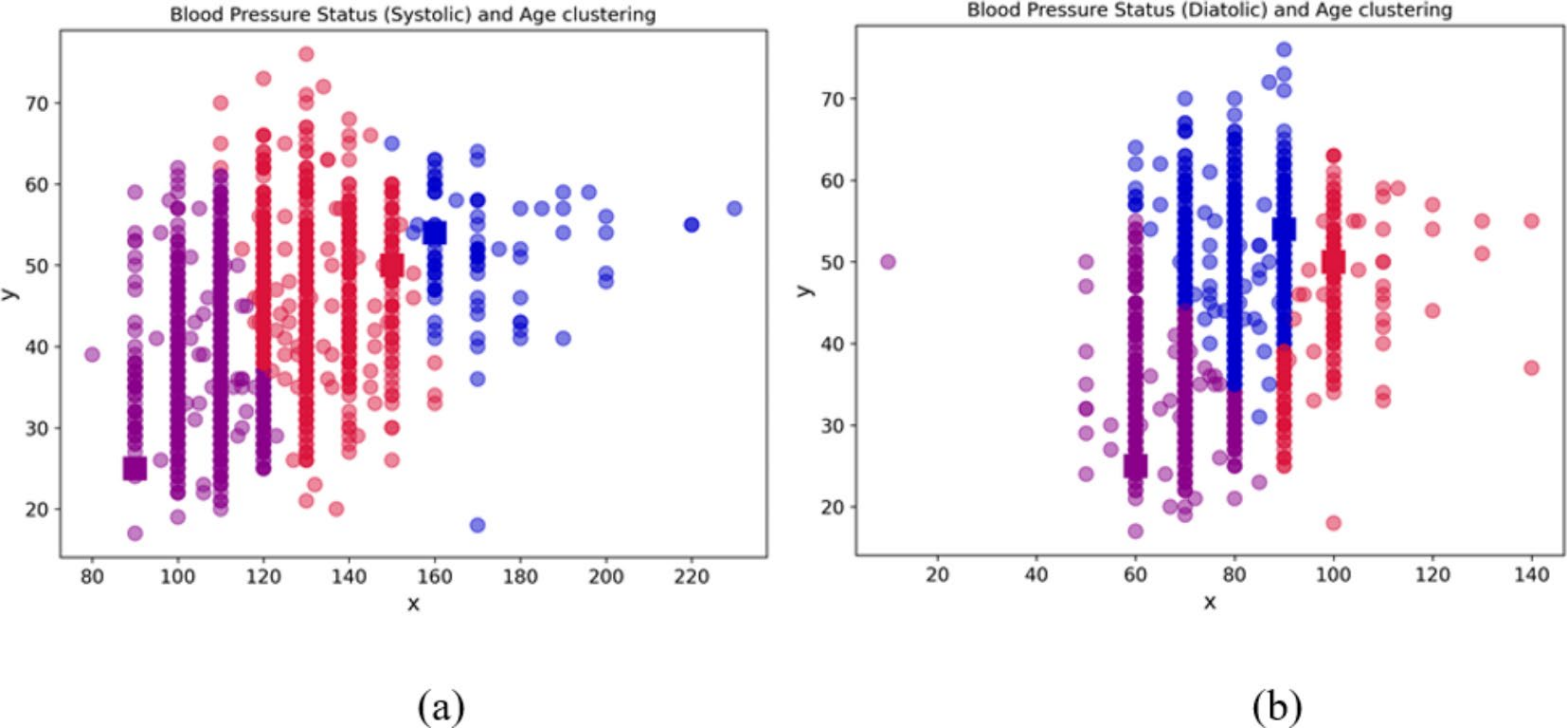
Clustering based on (**a**) blood pressure status (systolic) and age; (**b**) blood pressure status (diastolic) and age.

**Fig. 6:**
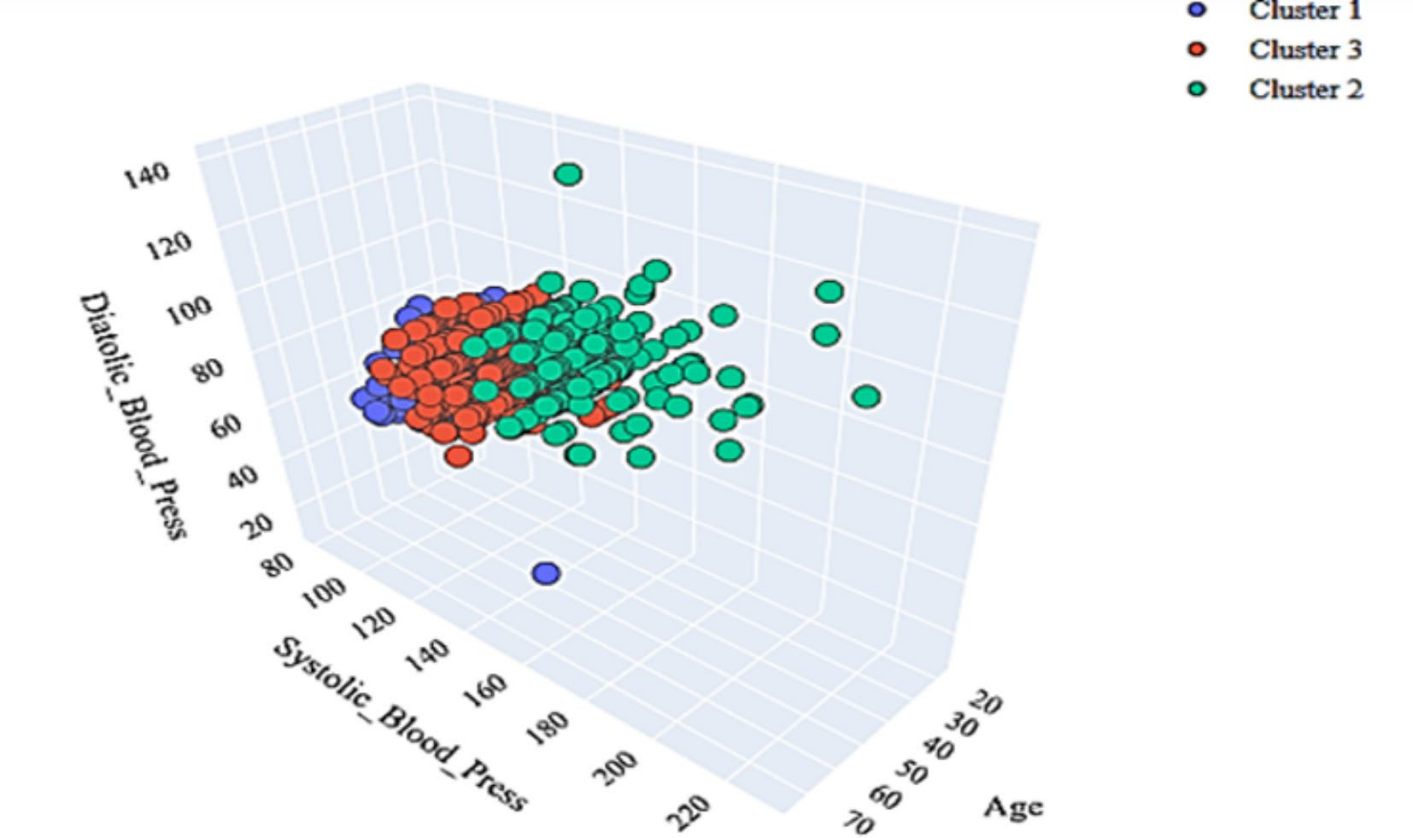
Clustering in 3D.

The application of the k-means algorithm as depicted in Fig. 5 shows a clustering of the workforce based on three major parameters: systolic, diastolic, and age. While Fig. 6 shows the clustering in three dimensions.

## Discussion

This study highlights the promising potential of leveraging machine learning techniques in workplace screening programmes for hypertension among university workforces. In this retrospective analysis of our university workforce health records using k-Means Clustering, we observed that the mean age for this working class was observed to be 42 years old. Similarly, we discovered that hypertension was prevalent among members of staff above the age of 40 irrespective of their gender or professional category (academic or non-academic). The analysis also revealed that there was a steady decline in the prevalence of hypertension from 2018 to 2022. We particularly found using k-Means Clustering relevant and appropriate for this analysis.

The average age for the working population in this analysis was observed to be 42 years. Evidence on the benefits of screening programmes for hypertension in adults in this age bracket is well-established. The United State Preventive Services Task Force (USPSTF) strongly recommends screening for high blood pressure in adults aged 18 years or older^21^. Similarly, the Canadian Task Force on Preventive Health Care continues to recommend screening for hypertension in adults aged 18 years and older without previously diagnosed hypertension^28^. Similar recommendations were made by the World Health Organisation and the International Society of Hypertension^28^. This makes the workplace an ideal setting for hypertension screening programmes and other health promotion interventions. It provides a convenient and accessible platform to reach many people who constitute a stable population and it may promote sustained positive peer support^30–32^.

Furthermore, we discovered that the prevalence of hypertension among members of staff increases with age and that hypertension prevalence is particularly high above the age of 40. This is not unusual because it is well-established in the literature that age is a predisposing factor for the development of primary hypertension^33^. A similar finding has been reported in another Nigerian study where age was found to be a risk factor for the high prevalence of hypertension among the urban population in Nigeria^34,35^. Given the earlier reported mean age of 40 for this working class, there is adequate evidence for the screening of employees for hypertension.

This analysis revealed that there was a steady decline in the prevalence of hypertension from 2018 to 2022 which implies that the screening programme helps to identify and better control blood pressure over the subsequent years. The reason for this is not far-fetched as this annual screening programme, carried out on staff members in the university community, serves as a regular sensitization mechanism put in place by the University, contributing to timely identification of undiagnosed hypertension and appropriate treatment of hypertension among the employees which promotes better blood pressure control. Similar benefits have earlier been attributed to workplace health promotion programmes^8,9,36^.

The consistent drop in the prevalence of hypertension from 2018 to 2022 observed in this study lays credence to the usefulness of k-Means Clustering for analysis of healthcare data from medical records. The relevance and reliability of k-Means Clustering for diagnosing hypertension has been earlier documented in literature. Tsoi et al. have shown that, among a number of Deep Learning and Machine Learning algorithms, K-means clustering produced the most consistent and dependable results for diagnosing hypertension^37^.

Limitations.

The study’s retrospective design nature limits the ability to establish causality between workplace interventions and changes in hypertension prevalence. However, the study’s longitudinal nature, spanning from 2018 to 2022, allows for the observation of trends over time, revealing a consistent drop in the prevalence of hypertension. Due to COVID-19 pandemic lockdown in Nigeria, the annual screening programme could not be conducted in 2020. Hence, there was no dataset on hypertension among the employees in 2020. Furthermore, since the employer bears the health care costs of the screening of the workforce in the setting of this study, there is a need for caution in the generalizability of the findings of this analysis to a very different context. Although the study was carried out in a single institution - limiting the generalizability of the findings to other settings or populations, the study includes a sizable dataset with 1,723 samples, increasing the reliability of the findings.

## Conclusion

We have shown with the use of machine learning techniques that a periodic workplace screening programme for hypertension is an effective, feasible, and sustainable strategy to diagnose and control hypertension among the working class. Workplace is as an idea setting for early detection and treatment of hypertension among the working class. Although the study demonstrates the feasibility of using machine learning for health assessments - illustrating the potential for advanced data analysis techniques to enhance health assessments in the workplace, it does not address the cost-effectiveness or practical implementation challenges of integrating these technologies into routine workplace health monitoring. Therefore, further research is needed to evaluate the barriers and facilitators of implementations of similar workplace health promotion programmes in the appropriate way.

## Electronic supplementary material

Below is the link to the electronic supplementary material.

Supplementary Material 1

## Data Availability

The datasets generated and/or analyzed during the current study are not publicly available because it involve data obtained from human participants. Dr. Olumide Adeleke should be contacted at (olumide.adeleke@bowen.edu.ng) for the dataset in this studyThe link to the data set https://data.mendeley.com/datasets/7d3kjdwn5d/1.
