# Supplementary material for: Machine learning evaluation of a hypertension screening program in a university workforce over five years
Source: Sci Rep. 2024 Dec 4;14:30255. doi: 10.1038/s41598-024-74360-1 (PMC11618500; doi:10.1038/s41598-024-74360-1)
Supplement: Supplementary file 1 — Supplementary Material 1 [file 41598_2024_74360_MOESM1_ESM.docx]

**Machine Learning Evaluation of a Hypertension Screening Program in a University Workforce over Five Years**

Olumide Thomas Adeleke^1,2^*, Segun Adebayo^3^, Halleluyah Aworinde^4^, Oludamola Victoria Adeleke^1^, Abidemi Emmanuel Adeniyi^4^, Oluwasegun Julius Aroba^5,6*^

^1^Directorate of Health Services, Bowen University, Iwo, Nigeria

^2^College of Health Sciences, Bowen University, Iwo, Nigeria

^3^College of Agriculture, Engineering and Science, Bowen University, Iwo, Nigeria

^4^College of Computing and Communication Studies, Bowen University, Iwo, Nigeria

^5^Honorary Research Associate, Department of Operations and Quality Management, Durban University of Technology, Durban, South.

^6^Centre for Ecological Intelligence, Faculty of Engineering and the Build Environment (FEBE), University of Johannesburg, Electrical and Electronic Engineering Science, Auckland Park Campus, Auckland Park, P.O. Box 524, Johannesburg 2006, South Africa.

*Correspondence: aroseglinks@yahoo.co.uk, [olumide.adeleke@bowen.edu.ng](mailto:olumide.adeleke@bowen.edu.ng)

**Abstract**

The global prevalence of hypertension continues excessively elevated, especially among low- and middle-income nations. Workplaces provide tremendous opportunities as a unique, easily accessible and practical avenue for early diagnosis and treatment of hypertension among the workforce class. The evaluation of such a Workplace Screening Strategy can give insight into its possible effects. Innovative machine learning approaches like k-means clustering are underutilized for such assessments. We set out to use this technology to analyze the results of our university's yearly health checkup of the employees for hypertension. An anonymized dataset including the demographics and blood pressure monitoring information gathered from workers in various departments/units of a learning organization. The overall amount of samples or data values is 1, 723, and the supplied dataset includes six attributes, such as year group (2018, 2019, 2021, 2022), Department/Unit (academic and non-academic), and gender (male and female), with the intended output being the blood pressure status (low, normal, and high). The dataset was analyzed using machine learning approaches. In this longitudinal study, it was discovered that the average age for the workforce is 42. Similarly, it was revealed that hypertension was common among employees over the age of 40, regardless of gender or occupational type (academic or nonacademic). The data also found that there was a consistent drop in the prevalence of hypertension from 2018 to 2022. According to the study findings, the use of machine learning algorithms for periodic evaluations of workplace health status monitoring initiatives (particularly for hypertension) is feasible, realistic, and sustainable in diagnosing and controlling hypertension among those in the workforce.

Keywords— Machine learning, Artificial Intelligence, ***Hypertension/diagnosis,*** Hypertension/prevention & control, ***Screening Programmes***, Occupational Health, Medical Records Systems, Workplace.

**1.** **INTRODUCTION**

Hypertension, simply known as elevated blood pressure, is the number one risk factor for death globally, significantly increasing the risk of developing cardiovascular, brain, and kidney diseases [1]. Globally, 1.28 billion people between the ages of 30 and 79 were reported to have hypertension in 2019. Two-thirds of these adults reside in low- and middle-income nations [2]. Regrettably, the WHO African Region has the highest prevalence of hypertension (27%) while the WHO Americas Region has the lowest prevalence of hypertension (18%) [2]. Sadly, an estimated 46% of persons with hypertension are ignorant of their disease and only 21% of persons with hypertension have it under control [3]. Screening programmes for hypertension could help to reduce morbidity and mortality associated with the condition in adults [4].

Employee's health status is of great importance to the stability and development of any institution and the society at large. However, it has been reported that the cardiovascular health status of the occupational population worldwide is not optimal [5]. Fortunately, workplace health programmes such as employee's periodic screening have been shown to protect the individual employee's overall health [6], improve his/her productivity [7], lower the overall medical costs [7], and reduce disease prevalence in general [8, 9]. Occupational screening of employees at work is an effective approach of identifying undiagnosed hypertensive people [10].

Artificial intelligence (AI) has been used in many areas of healthcare services, including medical imaging and diagnostics, pandemic management, virtual patient care, promoting medication and vaccine innovation, lowering the administrative burden on medical staff, tracking patient adherence to treatment regimens, conducting gait analyses for technology-assisted rehabilitation, and increasing patient engagement [11]. AI has also been used in some published research to diagnose [12-13] and to forecast the prevalence of hypertension [14-15] in the general population. However, machine learning approaches such as k-means clustering are underutilized in the context of workplace screening. Leveraging machine learning in workplace offers a promising opportunity for advanced data analysis techniques to enhance health assessments in the workplace.

K-Means Clustering is a machine-learning technique capable of providing useful insight into the behavior and patterns of data [16]. It has been applied in diverse areas in medical science such as cancerous cell detection, segmentation of brain images, skin treatment, intrathoracic airway trees, and abnormality detection of heart ventricles [17, 18]. Analysis of workplace and occupational screenings, where patient data may be complex, multidimensional, and subject to variable degrees of uncertainty, is particularly well-suited for k-Means clustering. In this study, we applied the k-Means Clustering algorithm to analyze health medical records from the university workforce [19].

**2. METHODOLOGY**

**2.1. Study Site and Participants**

This retrospective study was carried out between December 17, 2018, and December 20, 2022, at the Bowen University Hospital located in the urban setting of Iwo (population range of 250,000-499,999 inhabitants), Osun State in southwestern Nigeria. Bowen University, founded by the Nigerian Baptist Convention in 2001, is one of the oldest private coeducational institutions of higher education in Nigeria. This retrospective study included 1723 rows of datasets from the workforce at Bowen University across different academic and non-academic units of the university.

**2.2. Dataset Acquisition**

The dataset was collated from staff in different units of an academic institution for a period of four years (2018, 2019, 2021 and 2022). The blood pressure measurements used in this screening programme were taken by nurses and community health extension workers trained in manual blood pressure measurement, including proper body positioning during measurement. BPs were measured manually using a stethoscope and the appropriate sized brachial pressure cuff with a sphygmomanometer. The participants did not smoke or ingest caffeine or other stimulants or food in the 30 minutes before the measurements, which were taken after at least 5 minutes of rest in a quiet and calm environment Two BP readings were taken on both arms of employees in a sitting position and at 2-minute intervals. If the first two readings differ by more than 10mmHg, additional readings were obtained in line with American Heart Association (AHA) recommendations [20-22].

Equipment was inspected on a routine basis to ensure accuracy. Employees with measurements outside of the recommended range during the screening programme were advised to follow up with medical doctors at the Bowen University Hospital. High blood pressure (hypertension) at the time of screening is defined as a systolic blood pressure of 140 mmHg or higher or a diastolic blood pressure of 90 mmHg or higher National Heart, Lung, and Blood Institute [23]. We then anonymized these data from the university's yearly health checkup to ensure privacy while providing valuable insights into the prevalence of hypertension among different demographic groups.

**2.3. Data Preprocessing**

The dataset needs to be pre-processed before model training because each input sample contains different features with missing and inconsistent values. Most of the missing data in the dataset are measured values from staff, therefore, the study employed data-cleaning techniques. This entails locating and fixing mistakes or discrepancies in the data, including duplicates, outliers, and missing numbers [24, 25]. The data cleaning was accomplished with a variety of methods, including imputation, removal, and transformation.

2.4. Data Correlation

[Data mining task](https://www.sciencedirect.com/topics/computer-science/data-mining-task) play an important role in discovering patterns in data. This study employed feature correlation and k-means clustering to find relevant and non-redundant features in the data. Pearson correlation coefficient was employed to understand the linear relationships between variables. The Pearson Correlation Coefficient between two variables ***X*** and ***Y*** is computed as:

$$\rho_{\left( X,Y \right)}=\frac{COV(X,Y)}{\sigma_{X}\sigma_{Y}} (3)$$

Where cov is the covariance, $\sigma_{X}$is the standard deviation of variable X and $\sigma_{Y} is the standard deviation of variable$Y

2.5. Data Clustering

K-means clustering algorithms [30] is used to discover the structure of data and form the cluster. This is achieved by dividing the dataset into clusters according to data similarity. The technique involves initially selecting ‘k’ features randomly from original dataset D, as initial cluster centres. Based upon the distance between the features and cluster mean, the most similar object is assigned to the cluster. New mean value is then calculated for each cluster. The latter step is repeated until there is no redistribution of features in any cluster.

**2.6. Ethical Consideration**

This study was conducted using de-identified data obtained from the health medical record unit of the University Hospital. Ethical approval was sought and obtained from the Directorate of Research and Strategic Partnerships of the University.

**3. RESULTS**

3.1. Initial Dataset Exploration

The dataset contains both sociodemographic and medical records. The data set is summarized in Table 1 based on different features. The dataset contains seven features which are stated as year, age, systolic and diastolic values, blood pressure status, department or unit, and gender. There are 1723 rows in the dataset and the mean age is 42.64 years old, mean systolic and diastolic measurement are 120.91 and 78.4 respectively.

The dataset contains bio and blood pressure measurements values obtained from staff of different departments/ Unit in an academic institution. The total number of samples or data points are 1, 723 in which the input dataset contains six features, including year category (2018, 2019, 2021,2022), Department/Unit (academic and non-academic), gender (male and female), while the target output is the blood pressure status (low, normal and high) respectively.

**Table 1: Summarized Dataset**

| Statistic | Year | Age | Systolic_Blood_Press | Diatolic_Blood_Press | Blood_Press_Status | Dept_Unit | Gender | Category |
| --- | --- | --- | --- | --- | --- | --- | --- | --- |
| count | 1723 | 1723 | 1723 | 1723 | 1723 | 1723 | 1723 | 1723 |
| unique | NaN | NaN | NaN | NaN | 3 | 239 | 2 | 2 |
| top | NaN | NaN | NaN | NaN | Normal | Registry | M | Non-academic |
| freq | NaN | NaN | NaN | NaN | 1237 | 288 | 891 | 1172 |
| mean | 2019 | 42.6 | 120.9 | 78.4 | NaN | NaN | NaN | NaN |
| std | 1.3 | 9.7 | 18.6 | 23.1 | NaN | NaN | NaN | NaN |
| min | 2018 | 17 | 80 | 10 | NaN | NaN | NaN | NaN |
| 25% | 2018 | 35 | 110 | 70 | NaN | NaN | NaN | NaN |
| 50% | 2019 | 42 | 120 | 80 | NaN | NaN | NaN | NaN |
| 75% | 2019 | 50 | 130 | 80 | NaN | NaN | NaN | NaN |
| max | 2022 | 76 | 230 | 910 | NaN | NaN | NaN | NaN |

The number of individuals with normal blood pressure is higher than low or high blood pressure as shown in figure 1. The mean for systolic blood pressure value is about 124. Insight from figure 1a and b show that high blood pressure is prevalent among staff above the age of 40 irrespective of their gender or category (academic or non-academic).


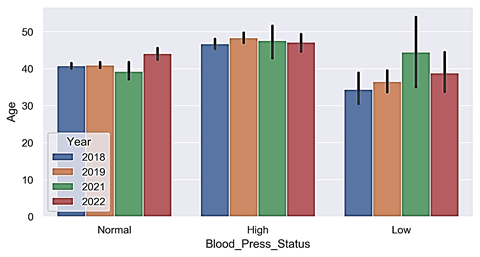

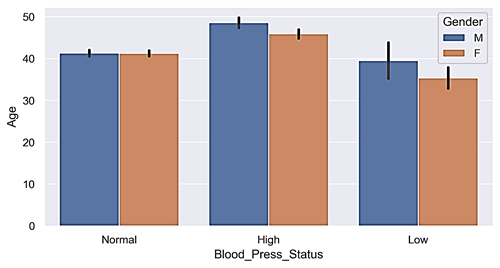


(a) (b)


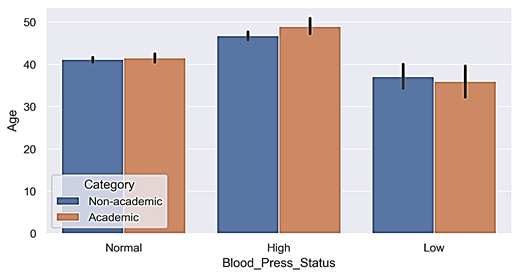


(c)

Figure 1: Blood Status against Age across different a) years, b) gender, c) category

Figure 2 shows the relationship plots of the dataset variables. The graphs revealed that there has been a relative decline in high blood pressure between 2018 and 2022. As against 29% high blood pressure in 2018, there has been a relatively significant fall to 18% in 2022 which accounts for 11% improvement in healthcare of individual staff by reason of blood pressure. Conversely, there seems to be a slight rise (3%) in low blood pressure from 2% in 2018 to 5% in 2022; this may be another trend to really pay attention to and the attendant contributive factors.

From the age-range variable, high blood pressure is prevalent among the age range 60 – 69 with 49% prevalent rate while the age group 20 – 29 has the lowest rate of 8%. In terms of low blood pressure, the age group 20 – 29 has the highest rate of 7% while the age group 40 – 49 has the lowest rate in low blood pressure of 1%.

For the staff category, the difference in high blood pressure rate is not so significant as there is only a difference of 1% between academic and non academic staff. Non academic staff members have a high blood pressure rate of 25% while the academic category of staff has 24%.


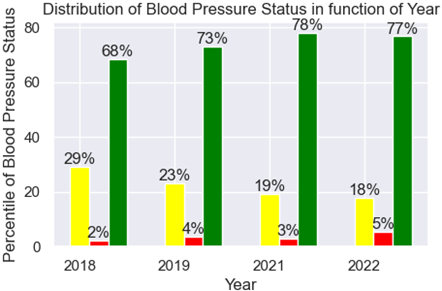

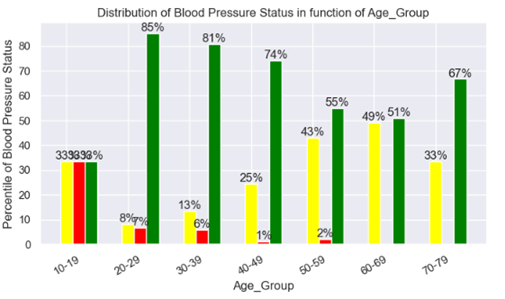


(a) (b)


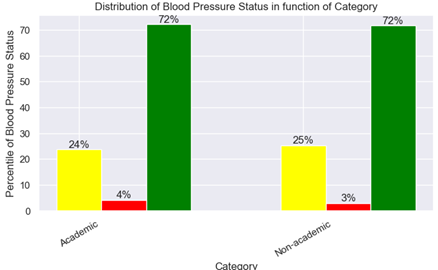


(c)

Legend


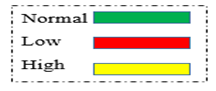


Figure 2: Relationship plots of the variables in the dataset: a) Distribution of blood pressure status in function of year; b) Distribution of blood pressure status in function of Age-group; c) Distribution of blood pressure status in function of categories

3.2. Features Correlation

After looking at the trends for the main variables in the previous section, the study considered the potential correlations between various variables. Figure 3 shows the Pearson Correlation matrix with significant values obtained from the dataset. Significant values have been identified as values with a 95% confidence and different from zero [29]. However, studies have shown that variable correlation can be full of noise or misrepresentation. To prevent such in our results, the study performed t-test [30] and the result is presented in figure 4. This shows that there is a strong correlation between systolic and age as well as diastolic and age.


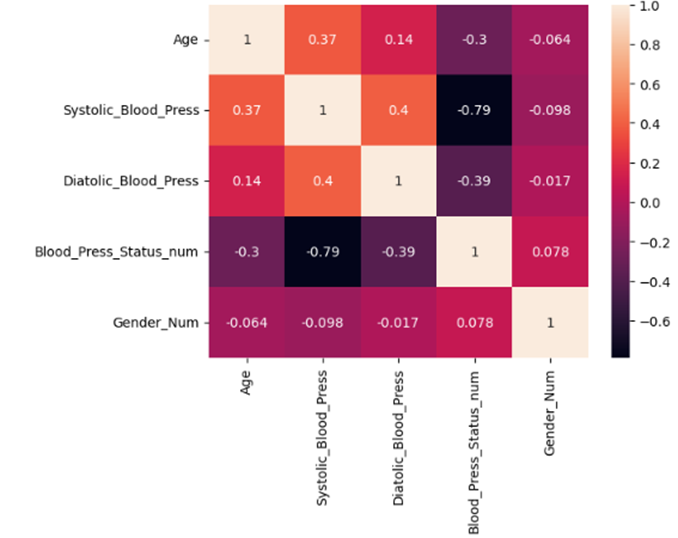


Figure 3. Pearson Correlation Matrix of the Variables in the dataset


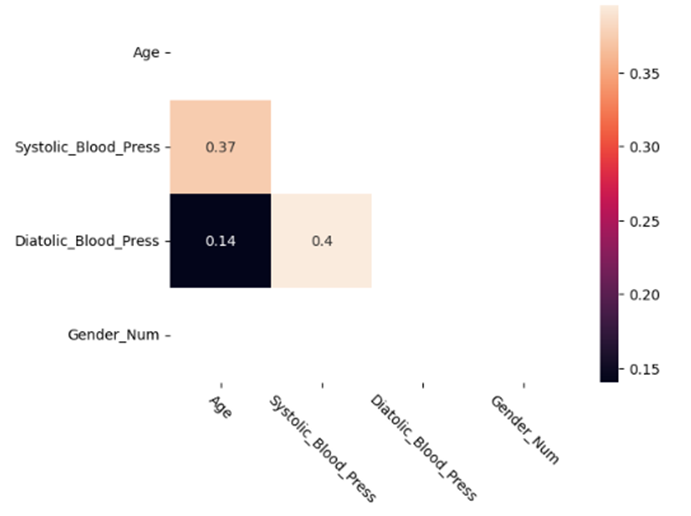


Figure 4. Pearson Correlation Matrix of the Variables in the dataset with non-significant values and the upper triangular filtered out

3.3.Insight through Unsupervised learning: Clustering

The application of the k-means algorithm as depicted in Figure 5 shows a clustering of the workforce based on three major parameters: systolic, diastolic, and age. While figure 6 shows the clustering in three dimensions.


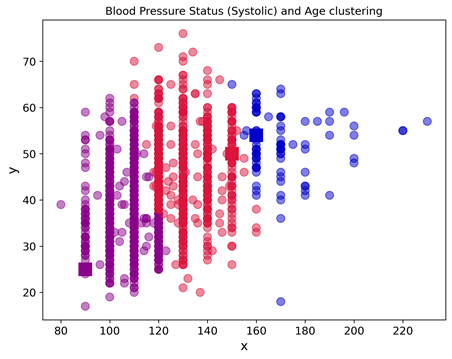

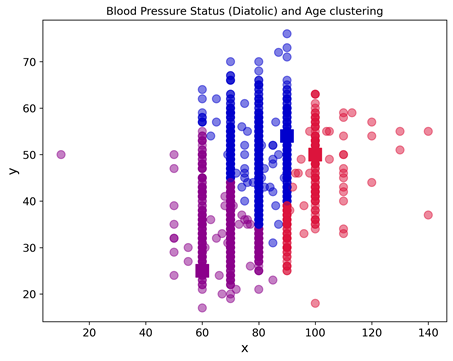


(a) (b)

Figure 5. Clustering based on a) blood pressure status (systolic) and age; b) blood pressure status (diastolic) and age


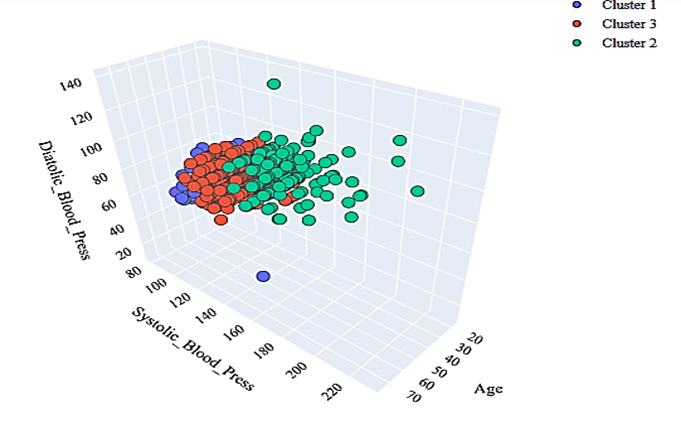


Figure 6. Clustering in 3D

**4. DISCUSSION**

This study highlights the promising potential of leveraging machine learning techniques in workplace screening programmes for hypertension among university workforces. In this retrospective analysis of our university workforce health records using k-Means Clustering, we observed that the mean age for this working class was observed to be 42 years old. Similarly, we discovered that hypertension was prevalent among members of staff above the age of 40 irrespective of their gender or professional category (academic or non-academic). The analysis also revealed that there was a steady decline in the prevalence of hypertension from 2018 to 2022. We particularly found using k-Means Clustering relevant and appropriate for this analysis.

The average age for the working population in this analysis was observed to be 42 years. Evidence on the benefits of screening programmes for hypertension in adults in this age bracket is well-established. The United State Preventive Services Task Force (USPSTF) strongly recommends screening for high blood pressure in adults aged 18 years or older [21]. Similarly, the Canadian Task Force on Preventive Health Care continues to recommend screening for hypertension in adults aged 18 years and older without previously diagnosed hypertension [28]. Similar recommendations were made by the World Health Organisation and the International Society of Hypertension [28]. This makes the workplace an ideal setting for hypertension screening programmes and other health promotion interventions. It provides a convenient and accessible platform to reach many people who constitute a stable population and it may promote sustained positive peer support [30-32].

Furthermore, we discovered that the prevalence of hypertension among members of staff increases with age and that hypertension prevalence is particularly high above the age of 40. This is not unusual because it is well-established in the literature that age is a predisposing factor for the development of primary hypertension [33]. A similar finding has been reported in another Nigerian study where age was found to be a risk factor for the high prevalence of hypertension among the urban population in Nigeria [34-35]. Given the earlier reported mean age of 40 for this working class, there is adequate evidence for the screening of employees for hypertension.

This analysis revealed that there was a steady decline in the prevalence of hypertension from 2018 to 2022 which implies that the screening programme helps to identify and better control blood pressure over the subsequent years. The reason for this is not far-fetched as this annual screening programme, carried out on staff members in the university community, serves as a regular sensitization mechanism put in place by the University, contributing to the timely identification of undiagnosed hypertension and appropriate treatment of hypertension among the employees which promotes better blood pressure control. Similar benefits have earlier been attributed to workplace health promotion programmes[8,9, 36].

The consistent drop in the prevalence of hypertension from 2018 to 2022 observed in this study lays credence to the usefulness of k-Means Clustering for analysis of healthcare data from medical records. The relevance and reliability of k-Means Clustering for diagnosing hypertension has been earlier documented in literature. Tsoi et al. have shown that, among a number of Deep Learning and Machine Learning algorithms, K-means clustering produced the most consistent and dependable results for diagnosing hypertension[37].

Limitations

The study's retrospective design nature limits the ability to establish causality between workplace interventions and changes in hypertension prevalence. However, the study's longitudinal nature, spanning from 2018 to 2022, allows for the observation of trends over time, revealing a consistent drop in the prevalence of hypertension. Due to COVID-19 pandemic lockdown in Nigeria, the annual screening programme could not be conducted in 2020. Hence, there was no dataset on hypertension among the employees in 2020. Furthermore, since the employer bears the health care costs of the screening of the workforce in the setting of this study, there is a need for caution in the generalizability of the findings of this analysis to a very different context. Although the study was carried out in a single institution - limiting the generalizability of the findings to other settings or populations, the study includes a sizable dataset with 1,723 samples, increasing the reliability of the findings.

**Conclusion**

We have shown with the use of machine learning techniques that a periodic workplace screening programme for hypertension is an effective, feasible, and sustainable strategy to diagnose and control hypertension among the working class. Workplace is as an idea setting for early detection and treatment of hypertension among the working class. Although the study demonstrates the feasibility of using machine learning for health assessments - illustrating the potential for advanced data analysis techniques to enhance health assessments in the workplace, it does not address the cost-effectiveness or practical implementation challenges of integrating these technologies into routine workplace health monitoring. Therefore, further research is needed to evaluate the barriers and facilitators of implementations of similar workplace health promotion programmes in the appropriate way.

**Ethical Approval and Consent to Participate**

The ethical clearance is attached to this submission.

We confirm that all methods were carried out in accordance with relevant guidelines and regulations.


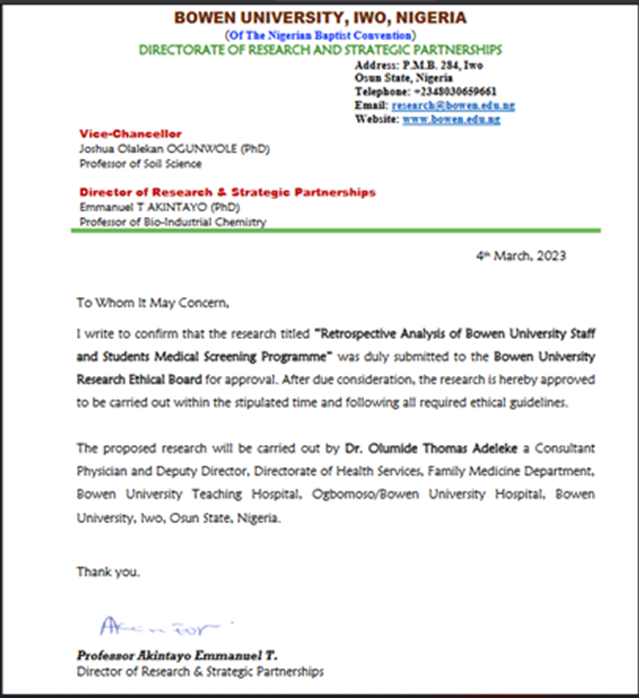


All informed consent was obtained from all subjects and/or their legal guardian(s).

**Consent for publication**

All the authors consent to submit and publish this research in the scientific report.

**Availability of supporting data**

The datasets generated and/or analyzed during the current study are not publicly available because it involves data obtained from human participants. Dr. Olumide Adeleke should be contacted at (olumide.adeleke@bowen.edu.ng) for the dataset in this study. The link to the dataset https://data.mendeley.com/datasets/7d3kjdwn5d/1

**Completing Interests**

All the authors declare that there is no competing interest in carrying out this research work. No complete interest in submitting the research work for publication.

**Authors Contribution**

Olumide Adeleke came up with the concept, methodology and Segun adebayo and Halleluyah worked on the modelling, Oludamola adeleke worked on the literature, Abidemi worked on Introduction, Oluwasegun Aroba worked on the abstract, summary, proofreading

References

[1] Chobanian AV, Bakris GL, Black HR, et al. Seventh report of the Joint National Committee on Prevention, Detection, Evaluation, and Treatment of High Blood Pressure. Hypertension. 2003 Dec. 42(6):1206-52.

[2] B. Zhou et al., “Worldwide trends in hypertension prevalence and progress in treatment and control from 1990 to 2019: a pooled analysis of 1201 population-representative studies with 104 million participants,” The Lancet, vol. 398, no. 10304, pp. 957–980, Sep. 2021, doi: 10.1016/S0140-6736(21)01330-1.

[3] S. Okello *et al.*, “Hypertension prevalence, awareness, treatment, and control and predicted 10-year CVD risk: a cross-sectional study of seven communities in East and West Africa (SevenCEWA),” *BMC Public Health*, vol. 20, no. 1, Dec. 2020, doi: 10.1186/S12889-020-09829-5.

[4] Legorreta AP, Schaff SR, Leibowitz AN, van Meijgaard J. Measuring the effects of screening programs in asymptomatic employees. Journal of occupational and environmental medicine. 2015 Jun 1;57(6):682-6.

[5] Zhao, X., Zhang, Y., Yang, Y., & Pan, J. (2022). Diabetes-related avoidable hospitalisations and its relationship with primary healthcare resourcing in China: A cross-sectional study from Sichuan Province. Health & Social Care in the Community, 30(4), e1143-e1156. doi: https://doi.org/10.1111/hsc.13522.

[6] Harden A, Peersman G, Oliver S, Mauthner M, Oakley A. A systematic review of the effectiveness of health promotion interventions in the workplace. Occupational medicine. 1999;49(8):540–8.

[7] Hu, J., Xu, T., Shen, H., Song, Y., Yang, J., Zhang, A.,... Chongqing, P. A. S. C. (2023). Accuracy of Gallium-68 Pentixafor Positron Emission Tomography–Computed Tomography for Subtyping Diagnosis of Primary Aldosteronism. JAMA Network Open, 6(2), e2255609. doi: 10.1001/jamanetworkopen.2022.55609.

[8] Jung H, Lee B, Lee JE, Kwon YH, Song H. Efficacy of a programme for workers with metabolic syndrome based on an e-health system in the workplace: a pilot study. Journal of telemedicine and telecare. 2012;18(6):339–43. 10.1258/jtt.2012.120318 . [PubMed] [CrossRef] [Google Scholar]

[9] Li, X., Liang, J., Hu, J., Ma, L., Yang, J., Zhang, A.,... On, B. O. T. C. (2024). Screening for primary aldosteronism on and off interfering medications. Endocrine, 83(1), 178-187. doi: https://doi.org/10.1007/s12020-023-03520-6.

[10] Seibt R, Hunger B, Stieler L, Stoll R, Kreuzfeld S. Early detection of undiagnosed hypertension based on occupational screening in the hotel and restaurant industry. BioMed Research International. 2018 Apr 8;2018.

[11]. Kuwaiti A, Nazer K, Al-Reedy A, Al-Shehri S, Al-Muhanna A, Subbarayalu AV, Al Muhanna D, Al-Muhanna FA. A review of the role of artificial intelligence in healthcare. Journal of personalized medicine. 2023 Jun 5;13(6):951.

[12]. López-Martínez F, Núñez-Valdez ER, Crespo RG, García-Díaz V. An artificial neural network approach for predicting hypertension using NHANES data. Sci Rep. 2020;10:10620.

[13]. Soh DCK, Ng EYK, Jahmunah V, Oh SL, San TR, Acharya UR. A computational intelligence tool for the detection of hypertension using empirical mode decomposition. Comput Biol Med. 2020;118:103630.

[14]. Ye C, Fu T, Hao S, Zhang Y, Wang O, Jin B, Xia M, Liu M, Zhou X, Wu Q, Guo Y, Zhu C, Li YM, Culver DS, Alfreds ST, Stearns F, Sylvester KG, Widen E, McElhinney D, Ling X. Prediction of Incident Hypertension within the Next Year: prospective study using Statewide Electronic Health Records and Machine Learning. J Med Internet Res. 2018;20:e22.

[15]. Kanegae H, Suzuki K, Fukatani K, Ito T, Harada N, Kario K. Highly precise risk prediction model for new-onset hypertension using artificial intelligence techniques. J Clin Hypertens. 2020;22:445–50.

[16] Carter, R.L. (2006) Solutions for Missing Data in Structural Equation Modeling. Research & Practice in Assessment, 1, 20-27. - References - Scientific Research Publishing.” Accessed: Sep. 30, 2023. [Online]. Available: <https://www.scirp.org/(S(lz5mqp453edsnp55rrgjct55.))/reference/referencespapers.aspx?referenceid=2792502>

[17] Zhu, C. (2024). Computational intelligence-based classification system for the diagnosis of memory impairment in psychoactive substance users. Journal of Cloud Computing, 13(1), 119. doi: https://doi.org/10.1186/s13677-024-00675-z

[18] Zhu, C. (2023). Research on Emotion Recognition-Based Smart Assistant System: Emotional Intelligence and Personalized Services. Journal of System and Management Sciences, 13(5), 227-242. doi: 10.33168/JSMS.2023.0515

[19] Wang, Q., Jiang, Q., Yang, Y., & Pan, J. (2022). The burden of travel for care and its influencing factors in China: An inpatient-based study of travel time. Journal of Transport & Health, 25, 101353. doi: https://doi.org/10.1016/j.jth.2022.101353

[20] Liu, J., et al., A comparative study of texture measures for human skin treatment. In the Proceedings of International Conference on Information, Communications, and Signal Processing. 1997. Singapore. pp. 170-174.

[21] Pal, N.R. and Pal, S.K., A review on image segmentation techniques. Pattern Recognition, 1993. 26(9): pp. 1277-1294.

[22] Pickering TG, Hall JE, Appel LJ, Falkner BE, Graves J, Hill MN, et al. Recommendations for blood pressure measurement in humans and experimental animals: part 1: blood pressure measurement in humans: a statement for professionals from the Subcommittee of Professional and Public Education of the American Heart Association Council on High Blood Pressure Research. Circulation 2005; 111:697-716

[23] Li, Q., You, T., Chen, J., Zhang, Y., & Du, C. (2024). LI-EMRSQL: Linking Information Enhanced Text2SQL Parsing on Complex Electronic Medical Records. IEEE Transactions on Reliability, 73(2), 1280-1290. doi: 10.1109/TR.2023.3336330

[24] Huang, Y., Wang, C., Zhou, T., Xie, F., Liu, Z., Xu, H.,... Xu, K. (2024). Lumican promotes calcific aortic valve disease through H3 histone lactylation. European Heart Journal, ehae407. doi: https://doi.org/10.1093/eurheartj/ehae407

[25] Yang, G., & Xiangming, L. (2024). Graduate socialization and anxiety: insights via hierarchical regression analysis and beyond. Studies in Higher Education, 1-17. doi: https://doi.org/10.1080/03075079.2024.2375563

[26] The Seventh Report of the Joint National Committee on Prevention, Detection, Evaluation, and Treatment of High Blood Pressure. US Department of Health and Human Services, National Institutes of Health; 2003. Available at: http://www.nhlbi.nih.gov/files/docs/ guidelines/jnc7full.pdf. Accessed December 20, 2023.

[27] Krist AH, Davidson KW, Mangione CM, Cabana M, Caughey AB, Davis EM, Donahue KE, Doubeni CA, Kubik M, Li L, Ogedegbe G. Screening for hypertension in adults: US Preventive Services Task Force reaffirmation recommendation statement. Jama. 2021 Apr 27;325(16):1650-6.

[28] Lindsay P, Gorber SC, Joffres M, Birtwhistle R, McKay D, Cloutier L. Recommendations on screening for high blood pressure in Canadian adults. Canadian Family Physician. 2013 Sep 1;59(9):927-33.

[29] Nugroho P, Andrew H, Kohar K, Noor CA, Sutranto AL. Comparison between the world health organization (WHO) and international society of hypertension (ISH) guidelines for hypertension. Annals of Medicine. 2022 Dec 31;54(1):837-45.

[30] Faculty of Public Health Medicine Committee on Health Promotion. Health Promotion in the Workplace. Guidelines for Health Promotion No. 40. London, UK: Royal College of Physicians, 1995.

[31] Tones K, Tilford S. Health Education: Effectiveness and Efficiency and Equity. London, UK: Chapman and Hall,1994.

[32] Isuezo SA. Systemic hypertension in blacks. An overview of current concepts of pathogenesis. Nigerian Postgraduate Medical Journal. 2003;10:144-53.

[33] Olatunbosun ST, Kaufman JS, Cooper RS, Bella AF. Hypertension in a black population: prevalence and biosocial determinants of high blood pressure in a group of urban Nigerians. Journal of human hypertension. 2000 Apr;14(4):249-57.

[34] Goetzel RZ, KahrTY, Aldana SG, Kenny GM. An evaluation of Duke University's Live for Life Health Promotion program and its impact on employee health. Am J Health Prom 1996; 10: 340-341.

[35] Benjamin DJ, Berger JO, Johannesson M, Nosek BA, Wagenmakers EJ, Berk R, Bollen KA, Brembs B, Brown L, Camerer C, Cesarini D. Redefine statistical significance. Nature human behaviour. 2018 Jan;2(1):6-10.

[36] Dhillon IS, Modha DS. Concept decompositions for large sparse text data using clustering. Machine learning. 2001 Jan;42:143-75.

[37]. Tsoi KKF, Chan NB, Yiu KKL, Poon SKS, Lin B, Ho K. Machine learning clustering for blood pressure variability Applied to systolic blood pressure intervention trial (SPRINT) and the Hong Kong Community Cohort. Hypertension. 2020;76:569–76.
